# Clinical application of sparse canonical correlation analysis to detect genetic associations with cortical thickness in Alzheimer’s disease

**DOI:** 10.3389/fnins.2024.1428900

**Published:** 2024-09-24

**Authors:** Bo-Hyun Kim, Sang Won Seo, Yu Hyun Park, JiHyun Kim, Hee Jin Kim, Hyemin Jang, Jihwan Yun, Mansu Kim, Jun Pyo Kim

**Affiliations:** ^1^Alzheimer’s Disease Convergence Research Center, Samsung Medical Center, Seoul, Republic of Korea; ^2^Department of Neurology, Samsung Medical Center, Sungkyunkwan University School of Medicine, Seoul, Republic of Korea; ^3^Neuroscience Center, Samsung Medical Center, Seoul, Republic of Korea; ^4^Department of Neurology, Seoul National University Hospital, Seoul, Republic of Korea; ^5^Department of Neurology, Soonchunhyang University Bucheon Hospital, Gyeonggi-do, Republic of Korea; ^6^Artificial Intelligence Graduate School, Gwangju Institute of Science and Technology, Gwangju, Republic of Korea

**Keywords:** Alzheimer’s disease, sparse canonical correlation analysis, genetics, cortical thickness, amyloid beta (Ab), single nucleotide polymorphism (SNP)

## Abstract

**Introduction:**

Alzheimer’s disease (AD) is a progressive neurodegenerative disease characterized by cerebral cortex atrophy. In this study, we used sparse canonical correlation analysis (SCCA) to identify associations between single nucleotide polymorphisms (SNPs) and cortical thickness in the Korean population. We also investigated the role of the SNPs in neurological outcomes, including neurodegeneration and cognitive dysfunction.

**Methods:**

We recruited 1125 Korean participants who underwent neuropsychological testing, brain magnetic resonance imaging, positron emission tomography, and microarray genotyping. We performed group-wise SCCA in Aβ negative (−) and Aβ positive (+) groups. In addition, we performed mediation, expression quantitative trait loci, and pathway analyses to determine the functional role of the SNPs.

**Results:**

We identified SNPs related to cortical thickness using SCCA in Aβ negative and positive groups and identified SNPs that improve the prediction performance of cognitive impairments. Among them, rs9270580 was associated with cortical thickness by mediating Aβ uptake, and three SNPs (rs2271920, rs6859, rs9270580) were associated with the regulation of *CHRNA2*, *NECTIN2*, and *HLA* genes.

**Conclusion:**

Our findings suggest that SNPs potentially contribute to cortical thickness in AD, which in turn leads to worse clinical outcomes. Our findings contribute to the understanding of the genetic architecture underlying cortical atrophy and its relationship with AD.

## 1 Introduction

Alzheimer’s disease (AD) is a progressive neurodegenerative disorder caused by the accumulation of β-amyloid (Aβ) plaques and neurofibrillary tangles, with subsequent neurodegeneration and cognitive decline (Serrano-Pozo et al., 2011). The amyloid hypothesis of AD, which suggests that Aβ accumulations in the brain are a central event in disease pathology, remains a dominant theory of disease causation (Goate et al., 1991; Selkoe, 1991; Karran et al., 2011). Neurodegeneration is the downstream pathologic process of Aβ accumulation and can be detected by cerebral atrophy or hypometabolism. Although neurodegeneration is not an AD-specific process, it is closely associated with its clinical symptoms and prognosis. Therefore, neurodegeneration is still recognized as a nonspecific but important biomarker in the National Institute on Aging-Alzheimer’s Association (NIA-AA) criteria for AD diagnosis and staging (Jack et al., 2018).

Several imaging genetic association studies have identified genetic biomarkers associated with cortical atrophy that serve as typical surrogate markers for neurodegeneration (Bakken et al., 2011; Wolthusen et al., 2015; Kim et al., 2020; Brouwer et al., 2022). However, the genetic mechanisms underlying the cortical atrophy in AD are complex. Further genetic studies are necessary to elucidate the intricate genetic mechanisms that contribute to cortical atrophy in AD. In recent years, advanced imaging genetics approaches have utilized complex machine-learning models (Kim et al., 2017, 2021; Arslan, 2018; Jacobs and Voineskos, 2020). These approaches not only identify genetic variants that affect brain structure and functional activities, but also provide a comprehensive understanding of the genetic mechanisms underlying these disorders using multivariate algorithms (Sim et al., 2013; Fang et al., 2016; Lorenzi et al., 2016; Kim et al., 2022; Kong et al., 2023).

Over the past few decades, several multivariate studies have emerged that integrate multiple data modalities, including partial least squares, parallel independent component analysis, and canonical correlation analysis (CCA), which have been used for genetic imaging studies (Witten and Tibshirani, 2009; Chi et al., 2013; Pearlson et al., 2015; Beaton et al., 2016; Kim et al., 2022). CCA has been widely used in the field of imaging genetics as a multivariate approach (Chi et al., 2013; Du et al., 2016; Fang et al., 2016; Liu et al., 2017). The fundamental concept underlying CCA is transforming the embedding space to maximize the cross-correlation between two sets of data (Hotelling, 1992). Researchers have employed L1 regularization in CCA, referred to as sparse CCA (SCCA), to identify genetic variants and address overfitting issues commonly encountered in high-dimensional datasets (Waaijenborg et al., 2008; Chu et al., 2013; Fang et al., 2016).

Cortical thickness is one of the sensitive markers of cortical atrophy and global and regional abnormality of cortical thickness in AD compared with cognitive unimpaired subjects have been widely reported (Lerch et al., 2005; Du et al., 2007; Dickerson et al., 2009). The pattern of brain atrophy in AD is different from that observed in normal aging, and cortical atrophy due to aging may be locally severe as in AD (Pini et al., 2016). Numerous studies support that Aβ pathophysiology may function as a trigger/facilitator of downstream molecular pathways that leads to cortical neurodegeneration (He et al., 2018; Busche and Hyman, 2020; Hampel et al., 2021) and cortical thinning is regarded as an indicator of the burden of neurofibrillary tangles and plaques, and neuronal loss that are related to AD (Dickerson et al., 2009).

**FIGURE 1:**
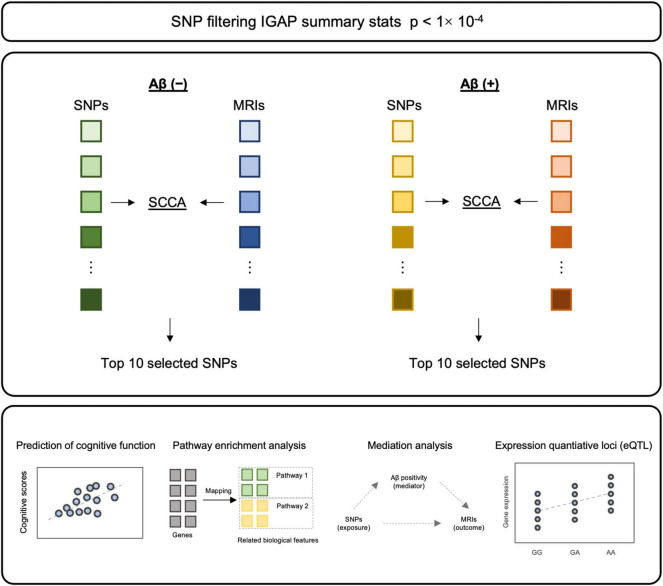
The schematic diagram of methodology. After filtering SNPs with *p*-value from IGAP summary statistics, SCCA was applied to each Aβ (–) and Aβ (+) for SNP selection. Top 10 SNPs from each model were selected, followed by validation and *post-hoc* analyses.

The purpose of this study is to identify genetic variants related to cortical thickness in the Korean population. In addition, we hypothesized that genetic mechanisms affecting cortical atrophy may differ depending on Aβ accumulation. Thus, we performed SCCA with genetic variants and cortical thickness independently in Aβ positive (Aβ (+)) and negative (Aβ (−)) and identified cortical thickness related SNPs in each group. Next, we evaluated whether selected single nucleotide polymorphisms (SNPs) contribute to improving the predictive performance of cognitive function. In addition, we performed functional effects of selected SNPs and evaluated the indirect effect of SNPs on the cortical thickness through Aβ (Figure 1).

## 2 Materials and methods

### 2.1 Study participants

The study participants were enrolled from the Korea-Registries to Overcome and Accelerate Dementia Research Project (K-ROAD). The K-ROAD aims to develop a genotype–phenotype cohort to accelerate the development of novel diagnostic and therapeutic techniques for Alzheimer’s and concomitant cerebrovascular diseases, and the 25 university-affiliated hospitals in South Korea have participated. All participants underwent neuropsychological testing, high-resolution T1-weighted magnetic resonance imaging (MRI), and microarray genotyping. The 1125 participants consisted of individuals diagnosed with dementia of Alzheimer’s type (DAT) (n = 447), amnestic mild cognitive impairment (aMCI) (n = 368), and the cognitively unimpaired (CU) (n = 310). All participants with CU met the following criteria: (1) no medical history that was likely to affect cognitive function based on Christensen’s health screening criteria; (2) no objective cognitive impairment in any cognitive domain on a comprehensive neuropsychological test battery (at least −1.0 SD above age-adjusted norms on any cognitive test); and (3) independence in daily living activities. All participants with aMCI met the criteria for aMCI with the following modifications (Albert et al., 2011): (1) subjective cognitive complaints by the participants or caregivers; (2) objective memory impairment below −1.0 SD on verbal or visual memory tests; (3) no significant impairment in daily living activities; and (4) non-demented status. The participants with dementia of the Alzheimer’s type met the NIA-AA criteria of probable AD (McKhann et al., 2011).

Participants with significant white matter hyperintensities (cap or band > 10 mm and longest diameter of deep white matter lesion > 25 mm); structural lesions, including cerebral infarction, intracranial hemorrhage, brain tumors, or hydrocephalus on MRI; abnormal laboratory results on complete blood count; electrolyte, vitamin B12, or folate levels; syphilis serology; or abnormal liver, kidney, or thyroid function tests were excluded from the study.

The Institutional Review Board of the Samsung Medical Center approved this study. Written informed consent was obtained from all the participants.

### 2.2 Genotyping and imputation

SNP data were genotyped using the Illumina Asian Screening Array BeadChip (Illumina, CA, USA). Quality control (QC) steps were performed using PLINK software (Purcell et al., 2007). The samples with call rate < 95%, sex-mismatch, excess heterozygosity rate (five standard deviations from the mean), and identify-by-descent ≥ 0.125 were excluded. The markers with call rate < 98%, minor allele frequency (MAF) < 1% and Hardy-Weinberg equilibrium *p* < 10^–6^ were excluded. After performing QC, un-genotyped markers were imputed using Minimac4 and reference haplotypes from HRC-r1.1 on the University of Michigan Imputation Server (Das et al., 2016). After performing imputation, SNPs with poor imputation quality r^2^ ≤ 0.8 and MAF < 1% were excluded. Finally, 4, 906, 407 biallelic SNPs in autosomal chromosomes (sex chromosome, mitochondrial, and pseudo-autosomal SNPs were excluded) were used for subsequent analyses.

### 2.3 Amyloid positron emission tomography (PET) acquisition and visual assessment

All participants underwent either ^18^F-florbetaben (FBB) or ^18^F-flutemetamol (FMM) PET at the SMC using a Discovery STe PET/CT scanner (GE Medical Systems, Milwaukee, WI, USA) in 3-dimensional (3D) scanning mode that examined 47 slices of 3.3 mm thickness spanning the entire brain (Jang et al., 2019). A visual assessment was performed to determine Aβ peptide deposition positivity, and the detailed process has been described previously (Cho et al., 2020). Briefly, tracer uptake was assessed according to the regional cortical tracer uptake system in four brain regions (frontal cortex, posterior cingulate cortex/precuneus, parietal cortex, and lateral temporal cortex) for FBB scans and in five regions (frontal, temporoparietal/insula, posterior cingulate/precuneus, lateral temporal, and striatum) for FMM scans. Aβ positivity was defined as whether tracer uptake was observed in any of these regions (Cho et al., 2020).

### 2.4 MRI acquisition and processing

Three-dimensional T1-weighted MR images were acquired using a 3.0T MRI scanner (Philips 3.0T Achieva; Philips Healthcare, Andover, MA, USA), as previously described (Kang et al., 2022). Images were processed using the CIVET anatomical pipeline to measure cortical thickness (Zijdenbos et al., 2002). The detailed processing pipeline has been described previously (Kang et al., 2022). Briefly, T1-weighted MR images were registered to the MNI-152 template (Collins et al., 1994) and corrected for intensity non-uniformities (Sled et al., 1998). Tissue classification was then performed (Zijdenbos et al., 2002), and the hemispheric inner and outer cortical surfaces were extracted using constrained Laplacian-based automated segmentation with a proximity algorithm (MacDonald et al., 2000; June et al., 2005). Cortical thickness was measured by calculating the Euclidean distance between the corresponding vertices on the inner and outer cortical surfaces (Lerch and Evans, 2005).

### 2.5 SNP identification based on SCCA

For genetic biomarker identification, we performed SCCA. Given datasets *X* ∈ ℝ^*n* × *p*^, *Y* ∈ ℝ^*n* × *q*^ with *n* samples, *X* denotes *p* features of SNP data, and *Y* denotes *q* features of imaging data. Witten et al. proposed the SCCA model. The model aims to identify the best association between two datasets, and is defined as follows:


$$\min_{u,v}-u^{T}\,X^{T}\,Y\,v$$

$$s.t.{||u||}_{2}^{2}=1,{||v||}_{2}^{2}=1,{||u||}_{1}\leq \tau_{1},{||v||}_{1}\leq \tau_{2},$$

where *u* and *v* denote the corresponding canonical vectors. The *l_1_* regularization was applied to control model sparsity (Witten and Tibshirani, 2009).

In our experiments, we first selected candidate SNPs and brain regions to mitigate overfitting. For the genotyping data, we filtered SNPs with *p* value < 1 × 10^–4^ in the International Genomics of Alzheimer’s Project (IGAP) summary statistics (Lambert et al., 2013), a meta-analysis of genome-wide association (GWA) data for AD. Additionally, we applied a clumping technique to prune redundant correlated effects resulting from linkage disequilibrium among the SNPs. For imaging data, the average cortical thickness in the temporal, frontal, parietal, and occipital lobes and the global mean cortical thickness adjusted for age, sex, education, and intracranial volume (ICV) were used. Then, we performed SCCA implemented in the Python package *‘cca-zoo’* (Parkhomenko et al., 2009) to identify genetic variants associated with cortical thickness. This analysis was performed separately for the Aβ (+) and Aβ (−) groups, as well as for the total samples. We selected a total of 20 SNPs (i.e., 10 SNPs from each of the Aβ (+) and Aβ (−) groups) in terms of the absolute weight of the canonical vectors for further analysis. The identified SNPs were used for subsequent analyses.

### 2.6 Validation of SNP selection model

The clinical efficacy of the identified SNPs was evaluated by examining their ability to predict global cortical thickness and cognitive function. First, the focus of the prediction tasks was to validate the clinical efficacy of the SNPs across different amyloid positivity groups (Aβ (+) and Aβ (−)). To accomplish this, the predictive models were trained for one group with repeated 5-fold cross-validation 10 times and tested on the other group. The elastic net regression was initially trained using the top 10 SNPs identified from SCCA within the Aβ (−) group and tested on the Aβ (+) samples. Subsequently, the model was trained using the same set of 10 SNPs from SCCA within the Aβ (+) groups and tested on Aβ (−) samples. Secondly, we evaluated the performance of SNPs in predicting cortical thickness and cognitive function for the Aβ (+) and Aβ (−) groups, as well as for the total samples, using elastic net regression.

### 2.7 Pathway analysis

We examined the biological concordance of specific SNPs identified through the aforementioned analysis by performing pathway analysis using Enrichr^1^ (Xie et al., 2021). For our pathway analysis, we utilized comprehensive and well-curated annotations provided by the Gene Ontology (GO) resources.^2^ By integrating information from the GO annotations, we aimed to gain deeper insights into the potential biological pathways and functional relationships implicated by the selected SNPs.

### 2.8 Statistical analysis

We performed statistical analyses to examine the impact of specific SNPs in two distinct aspects: (1) examination of the effects of the selected SNPs on AD (i.e., CU and AD) using logistic regression after controlling for age, sex, and education, and (2) examination of the effects of selected SNPs on cortical atrophy, with amyloid positivity as a mediator. Specifically, we employed structural equation modeling adjusted for potential confounding variables, including age, sex, education, and ICV, as implemented in R (*mediation package*). The significance of the results was determined using a *p*-value cutoff of < 0.05.

### 2.9 Expression quantitative trait loci (eQTL) analysis

We performed expression quantitative trait loci (eQTL) analysis to determine the functional effects of SNPs on gene expression using the GTEx database.^3^ To investigate whether any of the variants were eQTLs in brain tissues and whole blood, we used GTEx Analysis Release V8 (dbGaP Accession phs000424.v8.p2) with default parameters.

## 3 Results

### 3.1 Study participants

The demographic information and genotype characteristics of the participants are listed in Table 1. The age (mean [ ± standard deviation]) of the participants was 70.2 ( ± 8.5) years. The proportions of female and APOE ε4 carriers were 58.4% and 44.6%, respectively. Among 1125 participants, 647 (57.5%) were Aβ (+) and 478 (42.5%) were Aβ (−).

**TABLE 1:** Demographics of study participants.

	Total	Aβ−	Aβ +	*p-*value
N	1125	478	647	
Age, mean (SD)	70.2 (8.5)	70.7 (7.8)	69.8 (9.0)	9.55 × 10^–2^
Sex (female / male)	658 / 467	275 / 203	383 / 264	6.18 × 10^–1^
*APOE* ε4 count (0/1/2)	635 / 387 / 103	379 / 92 / 7	256 / 295 / 96	4.04 × 10^–41^
Diagnosis (CU / aMCI / DAT)	310 / 368 / 447	255 / 173 / 50	55 / 195 / 397	6.89 × 10^–84^
MMSE, mean (SD)	24.0 (5.5)	26.8 (3.6)	22.0 (5.7)	9.74 × 10^–59^
CDR-SB, mean (SD)	2.9 (3.1)	1.4 (1.9)	4.0 (3.3)	9.73 × 10^–81^

CU, cognitively unimpaired; aMCI, amnestic mild cognitive impairment; DAT, dementia of Alzheimer’s type; Aβ, amyloid beta. Note: The *APOE* ε4 count represents the number of ε4 copies in rs429358 and rs7412 single nucleotide polymorphisms.

### 3.2 SNP identification based on SCCA

The SNP data were filtered based on the *p* value obtained from the summary statistics of the IGAP (Lambert et al., 2013). Furthermore, linkage disequilibrium clumping was performed, resulting in the selection of 344 uncorrelated SNPs. The selected SNPs were used for subsequent analyses (Supplementary Table 1). The cortical thickness of 12 ROIs and cortical thickness after adjusting for covariates are displayed in Supplementary Figure 1. Both measures showed a significant difference between Aβ (+) and Aβ (−) groups, with *p* values < 0.001.

**FIGURE 2:**
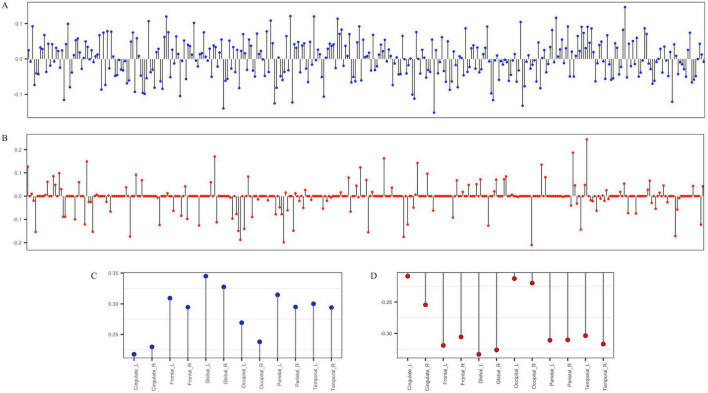
The stem plot of SNPs and regions of interest (ROIs) weights estimated by group-wise SCCA. **(A,C)** are weights of 344 SNPs and cortical thickness ROIs in the Aβ (–) group. **(B,D)** are the weights of 344 SNPs and cortical thickness ROIs in the Aβ (+) group. The y-axis represents the weights of variables, and the x-axis represents SNP indices **(A,B)** or the names of ROIs **(C,D)**.

Next, we used SCCA to select genetic variants associated with cortical thickness. The SCCA has been trained for the Aβ (−) and Aβ (+) groups and evaluated in terms of canonical correlation coefficients. The canonical correlation coefficients of those selected variants were 0.55 and 0.45 in the Aβ (−) and Aβ (+) groups, respectively. For the Aβ (−) group, we identified rs6743470 located near the *BIN1* gene on chromosome 2 (i.e., a canonical weight of −0.13), and rs141622900 located near the *APOC1* gene on chromosome 19 (i.e., a canonical weight of −0.14) as associated with imaging features. For the Aβ (+) group, rs157580 located near the *TOMM40* and rs7550917 and rs9270850, located near the *HLA-DQA1* gene, have been identified as genetic variants associated with imaging features. Detailed canonical loading weights and identified SNPs are shown in Figure 2 and Table 2. Among the top 20 SNPs selected from the two groups, five (rs73281586, rs6859, rs35879138, rs157580, and rs141622900) showed marginal associations with CU-AD diagnosis in our cohort (*p* < 0.05, Supplementary Table 2). The SCCA was trained on the total samples, and the canonical correlation was 0.42. Detailed canonical loading weights and identified SNPs are shown in Supplementary Figure 2 and Supplementary Table 3.

**TABLE 2:** Top 10 SNPs and canonical weights in SCCA.

	SNP	CHR	BP	Gene	Weight
Aβ −	rs4622634	19	1043864	ABCA7	−0.15
	rs77988388	6	27620034	RP1-15D7.1 (nearest)	0.15
	rs141622900	19	45426792	APOC1P1 (nearest)	−0.14
	rs6743470	2	127868435	BIN1 (nearest)	−0.13
	rs17612068	5	141883061	AC005592.2	−0.13
	rs1949100	5	118136826	CTC-448D22.1 (nearest)	−0.12
	rs190982	5	88223420	MEF2C-AS1	0.12
	rs858952	2	50875879	NRXN1	−0.12
	rs2271920	8	27316117	PTK2B	0.12
	rs12067173	1	41083947	RIMS3 (nearest)	0.12
Aβ +	rs7550917	1	193634651	RP11-21J7.1 (nearest)	0.24
	rs6859	19	45382034	PVRL2	−0.21
	rs17878252	19	46234155	FBXO46	−0.20
	rs157580	19	45395266	TOMM40	0.19
	rs73281586	7	26272643	CBX3 (nearest)	0.19
	rs35879138	19	45383139	PVRL2	−0.18
	rs115675626	6	32669833	MTCO3P1 (nearest)	−0.17
	rs9270850	6	32570717	HLA-DRB1 (nearest)	−0.17
	rs1328194	1	193702274	U3 (nearest)	0.17
	rs34141382	6	32608478	HLA-DQA1	0.16

SNP, single nucleotide polymorphism; CHR, chromosome; BP, base pair; Gene, mapped or nearest genes.

### 3.3 Prediction of AD biomarkers

To validate the efficiency of the SNPs, a model based on elastic net regression was constructed to predict AD biomarkers. First, we validated the efficiency of SNPs across different amyloid positivity groups (Aβ (+) and Aβ (−)). The prediction model employing the top 10 SNPs from SCCA within the Aβ (−) group showed high predictive performance for cortical thickness (r = 0.76) in Aβ (−) samples, whereas the low predictive performance of r = 0.18 in Aβ (+) samples. Similarly, the prediction model employing the top 10 SNPs from SCCA within the Aβ (+) group showed high predictive performance for cortical thickness (r = 0.50) in Aβ (+), whereas the low predictive performance of r = 0.36 in Aβ (−) (Table 3).

**TABLE 3:** Comparison of correlation coefficients (r) of predication models with top 10 SNPs for Aβ (+) and Aβ (−) groups.

	Train	Test
Design 1: train (Aβ −group) test (Aβ + group)	0.76 (0.75−0.77)	0.18 (0.17–0.19)
Design 2: train (Aβ + group) test (Aβ −group)	0.50 (0.48–0.52)	0.36 (0.33–0.39)

Correlation coefficients (r) and 95% confidence intervals (CI).

Subsequently, we compared the prediction performances of the models trained with the identified SNPs from SCCA and randomly selected SNPs. The prediction model with the top 10 SNPs (model 2) from SCCA within Aβ (−) group showed highest predictive performance in Aβ (−) samples (cortical thickness r = 0.72, MMSE r = 0.57, CDR-SB r = 0.36), whereas the model with randomly selected SNPs (model 3) (cortical thickness r = 0.68, MMSE r = 0.56, CDR-SB r = 0.35) and model with age, sex, and APOE ε4, ICV (model 1) (cortical thickness r = 0.67, MMSE r = 0.54, CDR-SB r = 0.31) exhibit relatively lower performance. Similarly, the prediction model employing the top 10 SNPs from SCCA within Aβ (+) showed the highest predictive performance in Aβ (+) samples compared to models 1 and 3 (Table 4). When evaluating the predictive performance in the total samples using the top 10 SNPs from each Aβ positivity group, for a total of 20 SNPs, the correlation coefficients were as follows: cortical thickness r = 0.44, MMSE r = 0.40, CDR-SB r = 0.31. In addition, the prediction performance of models with selected SNPs from total samples were shown in Supplementary Table 4. The prediction model employing the top 10 selected SNPs showed highest predictive performance.

**TABLE 4:** Correlation coefficients (r) of predication models for AD biomarkers.

	Cortical thickness	MMSE	CDR-SB
**Aβ −**			
Model 1	0.67	0.54	0.31
Model 2 (model 1 + top 10 SNPs)	0.72*^†^	0.57*	0.36*
Model 3 (model 1 +10 random SNPs)	0.68	0.56	0.35
**Aβ +**			
Model 1	0.36	0.33	0.20
Model 2 (model 1 + top 10 SNPs)	0.42*^†^	0.37*	0.24
Model 3 (model 1 +10 random SNPs)	0.38	0.36	0.27

Model 1 used age, sex, APOE ε4, and, if appropriate, ICV or education. Asterisks (*) represents the significant results when comparing the two correlation coefficients (model 1 and model 2) and dagger (†) represents the significant results when comparing the two correlation coefficients (model 2 and model 3). Statistical tests were performed to compare the two correlation coefficients using Hittner’s method (Hittner et al., 2003).

### 3.4 Pathway analysis

Pathway analyses were conducted using Enrichr, resulting in the enrichment of 134 and 106 gene sets in Aβ (−) and Aβ (+) groups, respectively. Table 5 presents the top 10 enriched gene sets in each group with a *q* value threshold of < 0.05. In the Aβ (−) group, the enriched gene sets included those associated with the regulation of synaptic transmission, postsynaptic potential, and dendrites. In the Aβ (+) group, enriched gene sets were related to MHC class protein binding and assembly. The results of gene set enrichment analysis for the selected SNPs in total samples were shown in Supplementary Table 5.

**TABLE 5:** Top 10 enriched pathway with the selected SNPs in Aβ (−) and Aβ (+) groups.

	Term	Ontology	OR	*P*-value
Aβ −	Positive regulation of synaptic transmission	BP	493.3	1.51 × 10^–5^
	Negative regulation of potassium ion transport	BP	444.0	1.81 × 10^–5^
	Positive regulation of synaptic transmission, glutamatergic	BP	221.9	6.31 × 10^–5^
	Positive regulation of excitatory postsynaptic potential	BP	221.9	6.31 × 10^–5^
	Modulation of excitatory postsynaptic potential	BP	211.3	6.91 × 10^–5^
	Regulation of amyloid-beta formation	BP	153.0	1.27 × 10^–4^
	Positive regulation of actin filament polymerization	BP	143.1	1.44 × 10^–4^
	Regulation of synaptic transmission, glutamatergic	MF	119.8	2.02 × 10^–4^
	Positive regulation of protein polymerization	BP	113.7	2.23 × 10^–4^
	Regulation of actin filament polymerization	CC	27.7	3.71 × 10^–4^
Aβ +	MHC class II receptor activity	MF	713.7	8.08 × 10^–6^
	MHC class II protein complex binding	MF	217.0	6.71 × 10^–5^
	Peptide antigen assembly with MHC class II protein complex	BP	416.2	2.04 × 10^–5^
	MHC class II protein complex assembly	BP	416.2	2.04 × 10^–5^
	Peptide antigen assembly with MHC protein complex	BP	312.1	3.43 × 10^–5^
	Immunoglobulin production involved in immunoglobulin-mediated immune response	BP	293.7	3.83 × 10^–5^
	MHC class II protein complex	CC	384.2	2.35 × 10^–5^
	MHC protein complex	CC	262.8	4.70 × 10^–5^
	Lumenal side of endoplasmic reticulum membrane	CC	199.7	7.85 × 10^–5^
	Antigen processing and presentation of exogenous peptide antigen via MHC class II	BP	208.0	7.27 × 10^–5^

BP, biological process; MF, molecular function; CC, cellular component; GO, gene ontology; OR, odds ratio.

### 3.5 Mediation analysis

**FIGURE 3:**
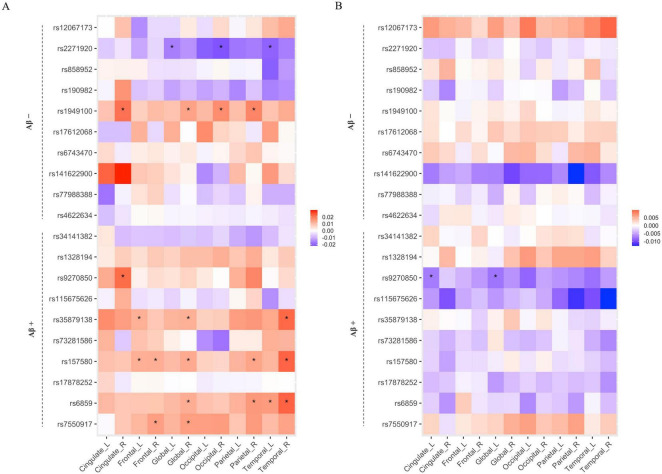
The results of mediation analysis for the relation of SNP with cortical thickness via Aβ positivity. The heatmaps of direct **(A)** and indirect **(B)** effects. The x-axis represents the name of ROIs, and the y-axis represents the selected SNPs from Aβ positive and negative groups. The associations with a *p*-value < 0.05 are indicated with an asterisk.

To assess the mediating effects of Aβ on the association between the identified SNPs and cortical atrophy, we performed a mediation analysis with Aβ positivity (Aβ (−) or Aβ (+)) as a mediator variable. Twenty SNPs (the top 10 SNPs in each amyloid positivity group) selected from SCCA were included in this analysis. Figure 3 shows that seven SNPs (rs2271920, rs1949100, rs9270850, rs35879138, rs157580, rs6859, and rs7550917) had significant direct or indirect effects on cortical thickness. Among these seven, rs9270850 selected from SCCA in the Aβ (+) group had significant indirect effects (Figure 3B).

### 3.6 eQTL analysis

**FIGURE 4:**
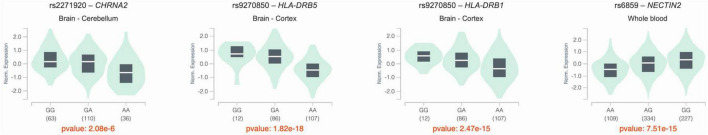
The violin plots of the eQTL results. The *x*-axis indicated the genotype of SNPs, and *y*-axis indicated the normalized expression levels of genes. All eQTL plots are downloaded from the GTEx portal.

We investigated whether the seven identified SNPs have eQTLs in brain tissues and whole blood using data from the GTEx eQTL database. Three of the seven SNPs, rs2271920, rs6859, and rs9270850, significantly affected the regulation of gene expression. The eQTL plots of the four SNPs were downloaded from the GTEx portal (Figure 4). The rs2271920 on chromosome 8 significantly regulated the expression of *CHRNA2* in the cerebellum (*p* value = 2.10 × 10^–6^). The rs9270850 significantly regulated *HLA-DRB5* (*p* value = 1.80 × 10^–18^) and *HLA-DRB1* (*p* value = 2.50 × 10^–15^) expressions in the brain cortex and rs6859 on chromosome 19 regulated the expression of *NECTIN2* in whole blood (*p* value = 7.50 × 10^–15^).

## 4 Discussion

In this study, we conducted an imaging genetic association analysis to identify the genetic biomarkers associated with cortical thickness. Our major findings are summarized as follows: First, through the implementation of the SCCA algorithm, we identified SNPs that demonstrated strong associations with cortical thickness. Second, our results indicate that the identified SNPs exhibit promising predictive performance for neurodegeneration and cognitive outcomes. This suggests their potential role as genetic markers associated with disease progression in AD. Third, our pathway enrichment analysis indicated that identified SNPs from the Aβ (−) and Aβ (+) groups were enriched for different gene sets. Additionally, one SNP identified in the Aβ (+) group exhibited associations with cortical thickness mediated by Aβ accumulation in the brain. These suggest that the genetic mechanisms that affect cortical atrophy may differ between the Aβ (−) and Aβ (+) groups. Overall, our findings highlighted the potential significance of the identified SNPs as novel genetic targets for understanding neurodegeneration in AD. These findings contribute to a growing body of research on genetic biomarkers associated with cortical thickness and their implications in AD pathology.

From the SCCA analysis, the global mean cortical thicknesses of the left and right hemispheres were the most prominent, followed by that of the frontal and parietal cortex. Global atrophy of the cortex, as well as regional atrophy, has been widely studied and demonstrated an abnormal reduction in patients with AD compared to cognitively normal control subjects (Braak and Braak, 1991; Lerch et al., 2005; Dickerson et al., 2009; Querbes et al., 2009). For the genetic data, we identified the top 10 SNPs for each Aβ (−) and Aβ (+) group. Twenty identified SNPs have been reported to be associated with the risk of clinically diagnosed AD in a European population (Lambert et al., 2013), but only five of them have a potential relationship with AD (p < 0.05). These results may be attributed to insufficient statistical power and ethnic differences. Recent genetic studies of AD have highlighted ethnic differences that may have affected our results.

We found that the identified SNPs improved the predictive performance for cortical thickness and cognitive function. Furthermore, we observed divergent patterns of these SNPs between the two groups. Specifically, the identified SNPs exhibited robust predictive capabilities for global cortical thickness in one group while facing challenges in achieving accurate predictions in the other group. These findings suggest that the genetic mechanisms affecting cortical atrophy may differ depending on amyloid accumulation. Our hypothesis was supported by the following observations: (1) The top 10 SNPs selected in each Aβ (−) and Aβ (+) group were not shared between groups. (2) The top 10 SNPs selected in one group showed poor predictive performance for cortical thickness and cognitive function in the other group. (3) These SNPs selected from the two groups were clustered into gene sets with different functional roles.

eQTL analysis revealed that these SNPs were significantly involved in the regulation of four genes (*CHRNA2*, *NECTIN2*, *HLA-DRB5*, and *HLA-DRB1*). Previous studies have reported that these genes are associated with AD pathophysiology. In particular, *CHRNA2* (cholinergic receptor nicotinic alpha 2 subunit) encodes neuronal acetylcholine receptor subunit alpha-2 (nAChRα2). Nicotinic acetylcholine receptors (nAChRs) are ligand-gated ion channels that produce neuronal receptors widely found in the central nervous system and are involved in synaptic transmission. *CHRNA2* is expressed in brain tissues and hippocampal CA1 region and previous studies have reported the effect of nAChRα2 on hippocampus-dependent learning and memory (Lotfipour et al., 2017) and plasticity of CA1 hippocampal synapses (Demontis et al., 2019). In addition, a variant of *CHRNA2* has been reported to be relevant to Alzheimer’s disease in the Chinese population (Ding et al., 2023), and *CHRNA2* is currently one of the targets of AD drug research (Xu et al., 2021). *NECTIN2* (nectin cell adhesion molecule 2) is expressed in astrocytes and neurons in the brain and plays important roles in the homeostasis of astrocytes and neurons and the formation of synapses (Mizutani et al., 2022). In addition, previous genetic association studies in European, Japanese, and African Americans reported that variations in *NECTIN2* are associated with AD (Harold et al., 2009; Takei et al., 2009; Logue et al., 2011), MCI to AD conversion in APOE ε4 non-carriers (Xiao et al., 2022), and cognitive trajectory (Rajendrakumar et al., 2024). Human leukocyte antigen (HLA) is a family of genes that encodes cell-surface proteins that play vital roles in immune system regulation (Shiina et al., 2009). The activities involved in immune responses, including infection, brain development, and plasticity, in AD pathogenesis may be determined by HLA genes (Wang et al., 2020). Moreover, the expression of *HLA-DRB1* and *HLA-DRB5* in microglia is positively correlated with measures of AD pathology (Mathys et al., 2019). Furthermore, many previous GWA and haplotype studies have suggested an association between variations in HLA genes and AD risk (Neill et al., 1999; Lambert et al., 2013; Mansouri et al., 2015; Zhang et al., 2022). *HLA-DRB1* has protective effects in APOE ε4 carriers against AD susceptibility (Ding et al., 2023), and CpGs of the *HLA-DRB5* gene are associated with AD pathological diagnosis (Yu et al., 2015). Although previous studies have reported associations between genes and the risk of AD, to date, no study has shown an association between the SNPs mapped to these genes and cortical thickness. Our findings can help achieve a better understanding of AD pathophysiology and uncover novel therapeutic targets for AD.

Pathway analysis revealed that genes identified in the Aβ (−) group were enriched for pathways related to dendrites, synaptic transmission, potassium ion transport, and Aβ formation. Meanwhile, genes identified in the Aβ (+) group were enriched for pathways related to the immune response, which are related to AD pathophysiology. Dendritic abnormalities in AD are widespread and occur in the early stages of the disease (Weigeldt, 1922; Cochran et al., 2014). Synaptic loss may lead to brain atrophy and the abnormalities in synaptic transmission and postsynaptic function are associated with cognitive decline (Ardiles et al., 2012; Subramanian et al., 2020; Tzioras et al., 2023). It is interesting that Aβ-related pathways were identified from SNPs identified in Aβ (−) group. This could be because subthreshold Aβ pathology is associated with worse pathological and clinical outcomes (Bischof and Jacobs, 2019). We also identified the potassium transport pathway. Potassium channels play an important physiological role in signaling mechanisms and are linked to the development of neurodegenerative diseases (Colom et al., 1998; Annunziato et al., 2002; Cordaro et al., 2022), and dysfunction of calcium-activated potassium ion channel activity is related to memory impairment (Trombetta-Lima et al., 2020). The peripheral and central immune systems are dysregulated in AD and are related to cognitive function and AD pathology (Bettcher et al., 2021; Wu et al., 2021). The pathological proteins of AD, such as Aβ peptides, can be swallowed by the microglia, which are presented to T lymphocytes after combination with particular HLA classes I and II. Furthermore, B lymphocytes secrete antibodies against Aβ peptides (Wang et al., 2020; Wu et al., 2021).

The strength of our study is the recruitment of participants using a standardized diagnostic protocol, including detailed neuropsychological tests, Aβ PET, and brain MRI. However, the present study had some limitations. We identified SNPs related to cortical thickness in AD using SCCA; however, the sample size was moderate. Although we used candidate SNPs to overcome the overfitting problem that leads to false-positive findings, replicating our findings in a larger independent dataset is still required. In addition, we used only a Korean population; further studies with racially diverse samples are needed to generalize our findings. Nevertheless, the fact that little research has been conducted on Asian populations makes the current study notable. In this study, we used candidate SNPs that showed higher associations with AD in the European population. However, these SNPs showed weaker associations in our study than in previous studies in the European population. Our findings highlight the importance of genetic association studies in diverse populations. In this study, we investigated the role of SNPs in neurodegeneration and Aβ neurological outcomes. However, because candidate SNPs were used, the genetic mechanisms of each pathway could not be fully elucidated.

In conclusion, using SCCA, we identified the SNPs that contributed to the prediction of cortical thickness in a Korean cohort. We investigated the involvement of these SNPs in AD by examining their association with cortical atrophy and Aβ-mediated cortical atrophy. AD is a complex multifactorial neurodegenerative disorder. Genetic association studies using AD biomarkers are needed to elucidate the complex genetic architecture of AD. Our findings contribute to our understanding of the genetic architecture underlying cortical atrophy and its relationship with AD.

## Data Availability

The original contributions presented in this study are publicly available. This data can be found here: https://www.ebi.ac.uk/eva/?eva-study=PRJEB72796.

## Footnotes

1
https://maayanlab.cloud/Enrichr/

2
http://geneontology.org

3
https://gtexportal.org
