# Supplementary material for: Clinical application of sparse canonical correlation analysis to detect genetic associations with cortical thickness in Alzheimer’s disease
Source: Front Neurosci. 2024 Sep 24;18:1428900. doi: 10.3389/fnins.2024.1428900 (PMC11458562; doi:10.3389/fnins.2024.1428900)
Supplement: Supplementary file 1 [file Data_Sheet_1.DOCX]

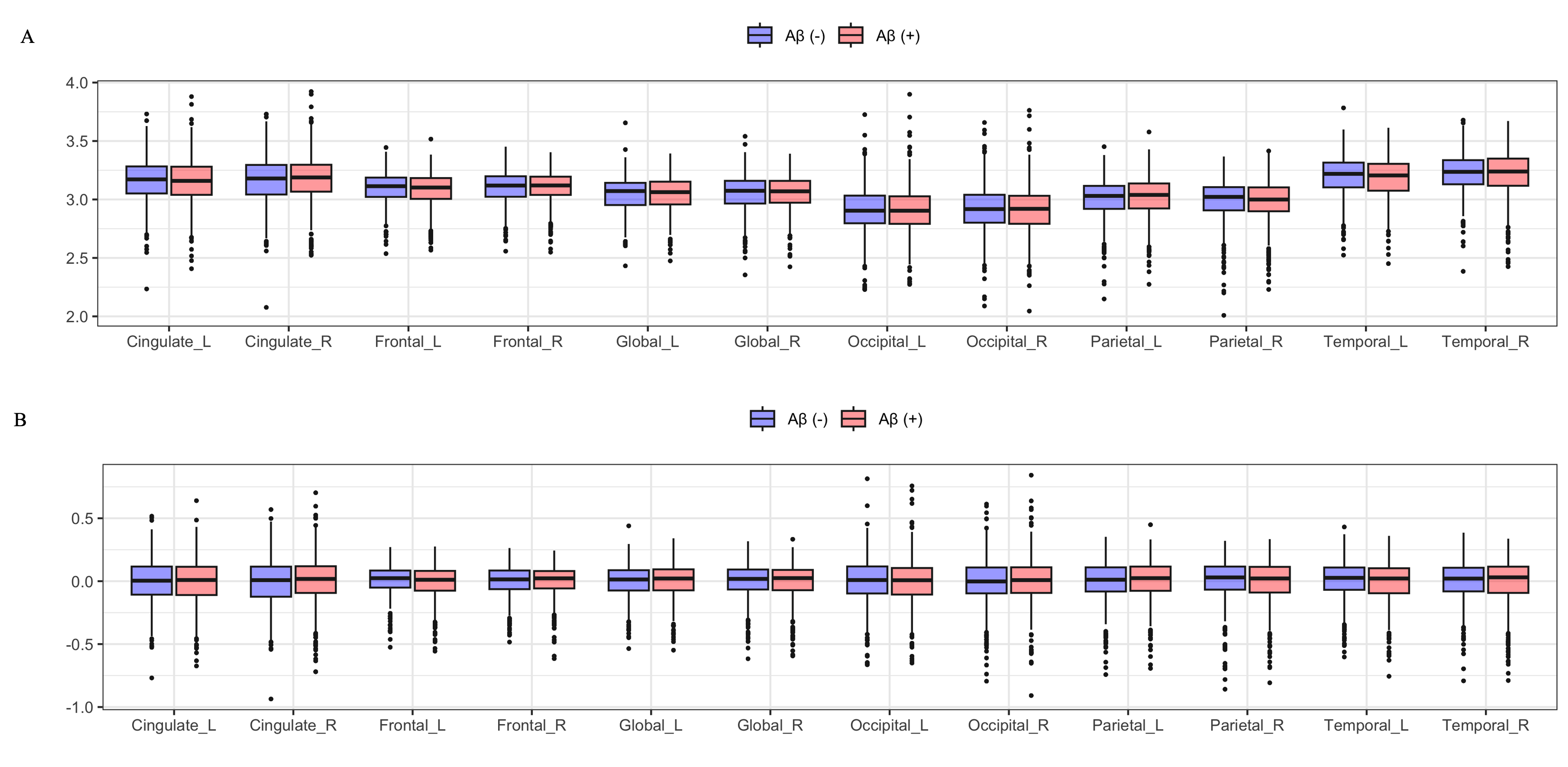


**Supplementary Figure S1.** Box plots of cortical thickness (upper) and cortical thickness after adjusting covariates (bottom) across Aβ positivity groups.


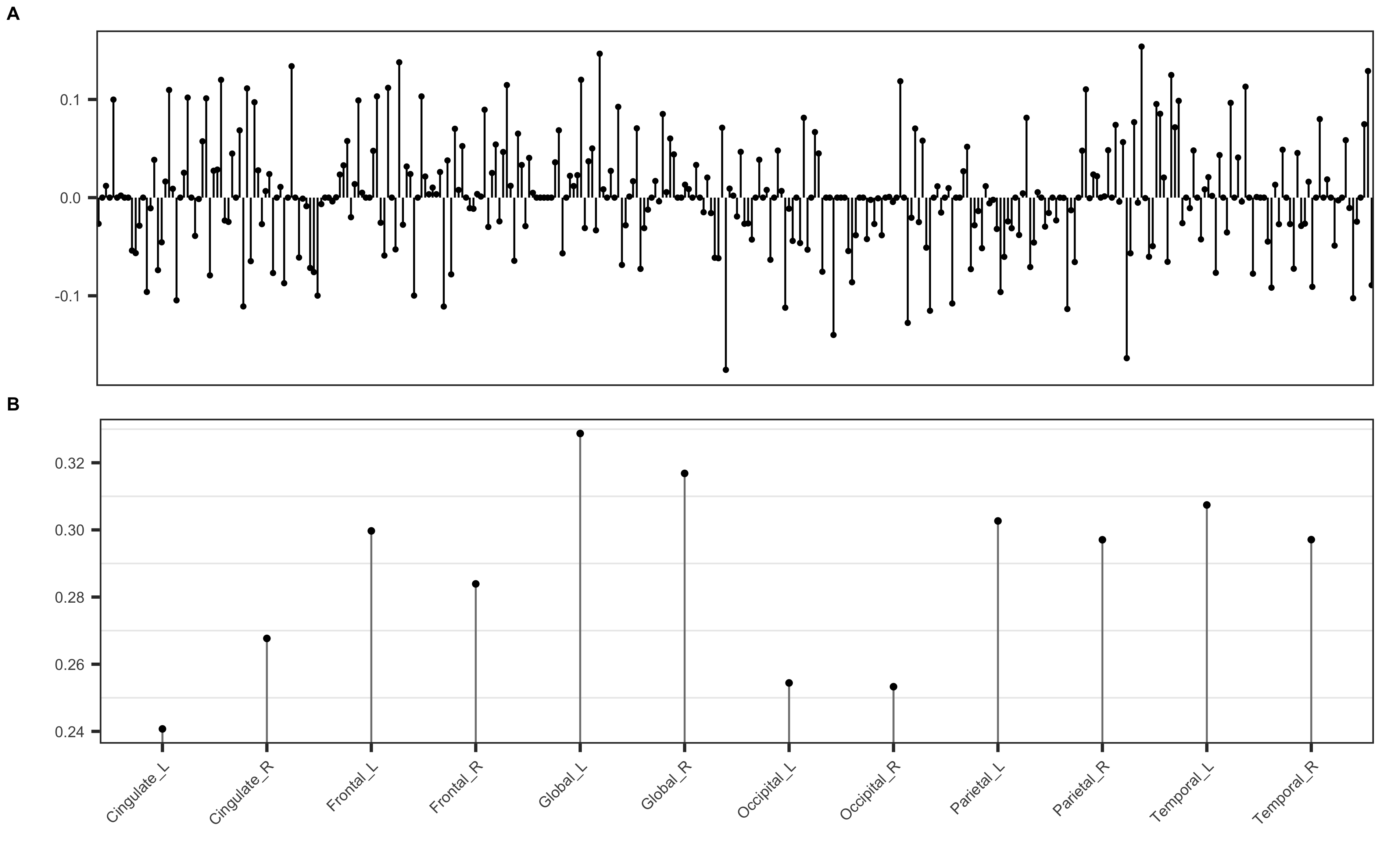


**Supplementary Figure S2.** The stem plot of SNPs and regions of interest (ROIs) weights estimated by SCCA with total samples.
